# Enhancing chronic low back pain management: an initial neuroimaging study of a mobile interoceptive attention training

**DOI:** 10.3389/fpain.2024.1408027

**Published:** 2024-09-30

**Authors:** Irina A. Strigo, Sergio Garcia Guerra, Salvatore Torrisi, Emily Murphy, Tiffany Toor, Veronica Goldman, Benedict J. Alter, An Thanh Vu, Rich Hecht, Jeff Lotz, Alan N. Simmons, Wolf E. Mehling

**Affiliations:** ^1^ Emotion and Pain Laboratory, San Francisco Veterans Affairs Health Care Center, San Francisco, CA, United States; ^2^ Department of Psychiatry, University of California San Francisco, San Francisco, CA, United States; ^3^ VA Advanced Imaging Research Center, San Francisco Veterans Affairs Health Care Center, San Francisco, CA, United States; ^4^ Department of Radiology and Biomedical Imaging, University of California San Francisco, San Francisco, CA, United States; ^5^ Osher Center for Integrative Health, University of California San Francisco, San Francisco, CA, United States; ^6^ Department of Anesthesiology and Perioperative Medicine, University of Pittsburgh Medical Center, Pittsburg, PA, United States; ^7^ Department of Orthopedic Surgery, University of California San Francisco, San Francisco, CA, United States; ^8^ Department of Psychiatry, University of California San Diego, San Diego, CA, United States; ^9^ San Diego Veterans Affairs Health Care Center, San Diego, CA, United States; ^10^ Department of Family and Community Medicine, University of California San Francisco, San Francisco, CA, United States

**Keywords:** interoceptive awareness, insula, nucleus accumbens, anticipation, mindfulness, MAIA

## Abstract

**Introduction:**

Chronic low back pain (cLBP) poses significant challenges, often addressed through avoidance or distraction. Emerging evidence suggests that mind-body interventions, like our novel Mind Your Pain (MyP) smartphone mobile application, may offer relief. We conducted a single-arm, mixed-methods neuroimaging study to assess the degree to which treatment response to our 8-week intervention, as measured by the reduction in the Pain, Enjoyment of Life and General Activity Scale (PEG), was associated with enhanced pain-related insula activation over time.

**Methods:**

Twenty-nine individuals with cLBP completed patient-reported assessments, qualitative sensory testing (QST) measures, and neuroimaging pre- and post-MyP. Functional MRI data during experimental heat pain on the left forearm were collected and analyzed, comparing responders (≥50% reduction in PEG scores) and non-responders.

**Results:**

MyP led to significant decreases in PEG scores overall. Furthermore, MyP responders exhibited increased pain-related activation in key brain regions, including the contralateral posterior insula, bilateral ventral anterior insula, ventral anterior cingulate, dorsolateral prefrontal cortex, and nucleus accumbens. Although baseline behavioral and sensory measures did not differ between the two responder groups, baseline neural differences related to the impact of the endogenous back pain were observed.

**Discussion:**

MyP appears to modify pain response and underlying neural circuitry, suggesting neural changes in interoception may serve as biomarkers for mind-body interventions in cLBP. This study highlights the potential of MyP as a novel approach for cLBP management, warranting further investigation.

## Introduction

Chronic pain, particularly chronic low back pain (cLBP), poses significant challenges, and current treatments, including medication and cognitive behavioral therapy (CBT), offer limited benefits (1–4). Rethinking/reframing and distraction from pain are core elements of CBT, with distraction useful for acute pain but of questionable benefit for chronic pain (5). Mind-body interventions like Mindfulness-Based Stress Reduction have shown modest success (6, 7), but understanding the neural mechanisms could enhance their effectiveness.

A key target of mind-body therapies is interoception (8–11), or the perception and representation of stimuli from the body that forms the sense of the physiological condition of the entire body, including pain (12–18). Various mind-body therapies directly involve interoception—specifically those referred to as contemplative practices, such as meditation, mindfulness-based approaches, yoga, and tai chi, that allot attention to the body (9). Further, various psychotherapy methods dealing with body-oriented healing, also involve interoception, such as Dialectical-Behavioral Therapy (19), Acceptance and Commitment Therapy (20), Somatic Experiencing (21), and Mindful Awareness in Body-oriented Therapy (22, 23). Additionally, there are mind-body techniques used specifically for interoception desensitization, such as the Mindfulness-based Interoceptive Exposure Task (MIET) which was developed within mindfulness-integrated cognitive behavioral therapy. The MIET has shown promising results in a pilot study with chronic musculoskeletal pain patients, demonstrating significant improvements in pain anxiety, duration, and intensity (24), as well as experimental pain (25), emphasizing the need for further study.

In the brain, the cortex's central hub for processing interoception is the insula cortex (IC), which is critical for the perception and modulation of acute pain (9), pain chronification and recovery (23). Pain, an affective and often unpleasant experience, transmits via lamina I spinothalamic pathway through a specific relay in the thalamus to the posterior insula (12). Its conscious perception involves integration with reward, exteroception, and top-down frontal cortex-associated appraisal in the middle and anterior insula (26). The associated motivation for an adaptive response, or homeostatic control of the brain and body, is similarly generated in the cingulate cortex (13, 27). Further, perception and cortical representation of the body are altered in those with cLBP (28, 29), including dysfunctional brain activity in the IC, and altered IC-ACC connectivity (30–32). Additionally, it has been found that patients with cLBP generally prefer distraction over interoceptive awareness through mindful attention to their pain (33). However, distraction, i.e., avoidance of experience in the present moment, is the opposite of mindful attention (34). Our published work relates distraction and avoidance to decreased insula responses to experimental pain (35, 36). We showed that reduced insula activity and IC-ACC connectivity modulate avoidant behavior in combat trauma victims with and without cLBP (36, 37). Interoceptive attention, or awareness of interoceptive sensations, goes along with IC activity opposite to distractive (and avoidant) thinking (38, 39). Only one neuroimaging study to date examined the effects of an interoception-based intervention, Mindful Awareness in Body-oriented Therapy, in healthy volunteers and found plasticity within insula activation (i.e., less interoception-related deactivation) and increased IC-ACC connectivity during an interoceptive breathing task (40). Modulating interoceptive circuitry with therapeutic approaches to potentially improve chronic pain management may be a promising direction for chronic pain research (41).

Here we report on a single-arm, mixed-methods neuroimaging study of an interoceptive attention training in individuals with cLBP administered through a smart-phone mobile application over 8 weeks. We aimed to examine the degree to which treatment response to our intervention, as measured by the reduction in the Pain, Enjoyment of Life and General Activity Scale (PEG), our primary outcome measure for response to treatment, was associated with enhanced pain-related insula activation over time. Based on the literature above we hypothesized that the proposed treatment would (1) produce increased self-reported mindful interoceptive awareness over time, (2) that this would be reflected in greater experimental pain-related brain activation within the insula over time, and (3) that experimental pain-related insula activation would relate to improved treatment.

## Materials and methods

### Study design

We conducted a single-arm, longitudinal brain imaging study in patients with cLBP to examine the effects of interoceptive attention training on the interoceptive neural circuitry induced by experimental pain. The interoceptive attention task was administered through an in-house designed mobile application *Mind Your Pain (MyP)*, developed by the investigators solely for research purpose (see below). At baseline (pretreatment) and follow-up (posttreatment) we applied standard pain self-report outcomes (PROs), quantitative sensory testing (QST; used for measuring thermal and pressure pain thresholds, temporal summation of pain and conditioned pain modulation), and functional magnetic resonance imaging (fMRI). The study protocol and initial self-reported quantitative outcomes, as well as the results from a qualitative analysis of exit interviews, are reported separately (42). Note that the sample reported here varies slightly from the qualitative analysis (42), as the current sample excludes two participants for MRI-related exclusions who continued with treatment and includes two additional participants enrolled after data collection for the qualitative analysis (42) was locked.

### Participants

Thirty participants (6 males, mean ± SD age: 49 ± 12, range 26–64) gave written informed consent to participate in this study, which was approved by the UCSF Human Research Protection Program and San Francisco Veterans Affairs Healthcare System Research and Development Committee (SFVAHS RDC), and registered with ClinicalTrials.gov (#NCT06186193). Study inclusions were: (1) English-speaking men and women aged 18–65 years old; (2) experiencing chronic back pain (cLBP) defined according to the NIH Research Task Force Recommendation on Research Standards for cLBP (43–45) and (3) demonstrating low levels of interoceptive awareness and habitual distraction from the Multidimensional Assessment of Interoceptive Awareness scales (MAIA-2), specifically using the non-distraction subscale (46). (See Supplementary Material for further details and study exclusions). The rationale for this inclusion criterium was that the MAIA assesses participants' common coping mechanism of habitual distraction and ignoring pain (labeled as Non-Distraction) (33), while the intervention teaches paying attention. We wanted to avoid a potential ceiling effect for participants who already are high at Non-Distraction at baseline. One female participant was excluded from the analyses due to extensive motion in the MRI scanner. The final sample reported here included 29 participants with cLBP (6M; age: 48.6 ± 12.2 years old) who completed pretreatment and post-treatment experimental sessions, which included PROs, QST, and MRI/fMRI.

### Primary outcome measure for treatment efficacy

The Pain, Enjoyment of Life and General Activity Scale (PEG) (47) is a 3-item scale measuring average pain intensity, interference with enjoyment of life, and interference with general activity over the past week, derived from the Brief Pain Inventory (BPI) (48). PEG scores are calculated as the mean of three 0–10 numerical rating scales. Changes in PEG score over the 8-week intervention (see below) were used to examine treatment response—this approach yields quick and interpretable result. Individuals were categorized into treatment responders (MyP+) and non-responders (MyP−) whereby at least 50% reduction in PEG scores served as the threshold for responder status. A large % reductions in PEG scores were used to indicate treatment response, ensuring that there was a clinically meaningful change and reducing the influence of non-specific effects of the treatment (49).

### Pain-anticipation fMRI paradigm

The pain-anticipation paradigm has been reported on previously (36, 50). Briefly, two predetermined temperatures, individually adjusted based on the subject's heat pain thresholds, were used to elicit low-pain and a high-pain sensation, respectively (mean ± SD thermal pain threshold: 44.55 ± 1.18°C; low-pain intensity: 22 ± 17.34; high-pain intensity: 40.49 ± 19.02, *p* < 0.0001). Stimulation was delivered through a 9 cm^2^ thermode (Medoc TSA2, Ramat-Yishai, Israel) on the participant's left forearm (50). Each trial began with a period of anticipation (10 s) initiated by a visual cue that was always followed by painful stimulation (7 s, either high-pain or low-pain), and a period of rest (jittered between 24 and 30 s) before the next trial began. Three types of visual cues were used: a red cross cuing high-pain, a green cross cuing low-pain and a yellow cross, cuing pain of uncertain intensity (at 50% probability being high or low, which was not known to the subject) (see Supplementary Material for details). The analysis of uncertain cues is beyond the scope of this work (51) and will be presented elsewhere.

### MRI image acquisition

Scans were performed using a 3 T Siemens Skyra at the San Francisco Veterans Affairs Medical Center with a 32-channel NOVA head coil. High-resolution T1-weighted MPRAGE structural scans (0.8 mm isotropic) and gradient echo B0 phase map acquisition (2 mm isotropic) matched the matrix of functional runs. Functional echo planar imaging (EPI) consisted of two runs with TR = 0.82 s, TE = 35 ms, flip angle = 58, and matrix = 104 × 104. Earplugs, head pads, and a knee pillow provided subject comfort and stability during the 10 min 18 s scans.

### fMRI image processing

#### Preprocessing and first-level modeling

Preprocessing and first-level modeling utilized afni_proc.py in AFNI (52) version 23.1.07. Structural T1w MPRAGE scans underwent nonlinear skull-stripping and registration to the 2009 MNI template (53, 54). Physiological noise removal involved tissue segmentation, head motion parameter regression, and subject motion criteria (55). Functional volumes underwent slice-timing correction, nonlinear warping to the MNI template, and smoothing with a 4 mm FWHM kernel. The task was modeled using a GLM with a standard (BLOCK) HRF and REML autocorrelation correction (56).

#### Group level modeling and statistical correction

To evaluate significance of effect of intervention, the whole brain linear mixed effects model with group (responder or MyP+, and non-responder or MyP−), time (pre, post) and stimulus (i.e., high-pain, low-pain) with subject as a random factor was run with AFNI function 3dlMEr (57) separately for pain anticipation and pain stimulation. Voxel-level results were thresholded at *p* = 0.005 and cluster volume at *p* < 0.05, using 3dFWHMx and 3dClustSim (58). The effects were examined for group-by-time interaction, and for baseline task and group effects. Furthermore, to better understand neural differences in response to MyP, we explored how the groups differed in neural substrates underlying their endogenous back pain. Two additional linear mixed effects models with group, PEG scores and stimulus were run on baseline activation for anticipation and stimulation with subject as a random factor using AFNI function 3dlMEr (57). We explored voxel level significance between *p* = 0.001 and *p* = 0.005 and cluster level significance of *p* < 0.05. Voxel-wise results were statistically corrected and visualized as recommended (59).

### Self-reported battery

A self-reported battery consisting of NIH HEAL common data elements (60) was collected with REDCap (Research Electronic Data Capture) (61) at baseline and at 8 weeks following completion of the app-based intervention (see below). The battery assessed pain catastrophizing (Pain Catastrophizing Scale (PCS) (62), pain anxiety (Pain Anxiety Symptoms Scale (PASS20) (63), perceived stress (Perceived Stress Scale (PSS4) (64), fear-avoidance (Fear-Avoidance Belief Questionnaire (FABQ) (65), chronic pain acceptance (Chronic Pain Acceptance Questionnaire (CPAQ) (66), pain self-efficacy (Pain Self Efficacy Questionnaire (PSEQ) (67), interoceptive awareness (Multidimensional Assessment of Interoceptive Awareness (MAIA2) (46), and mindfulness (Five Facet Mindfulness Questionnaire (FFMQ) (68). Expectation of Pain Relief due to treatment (69) and Patient Global Impression of Change (PGIC) were collected at baseline and at 8 weeks, respectively (see (70) and Supplementary Material for details; for additional self-reported questionnaires, see also (42). All measures were analyzed using their total score, as well as sub-scales where applicable. Importantly, this included all 8 sub-scales of the MAIA2, addressing 5 dimensions of body awareness: (1) Noticing (Awareness of Body Sensations), (2) Not-Distracting (Emotional Reaction and Attentional Response to Sensations), (3) Not-Worrying (Emotional Reaction and Attentional Response to Sensations), (4) Attention Regulation (Capacity to Regulate Attention), (5) Emotional Awareness (Awareness of Mind-Body Integration), (6) Self-Regulation (Awareness of Mind-Body Integration), (7) Body Listening (Awareness of Mind-Body Integration), & (8) Trust (Trusting Body Sensations) (43).

### Quantitative sensory testing (QST)

A modified protocol from the German Research Network on Neuropathic Pain (DFNS (71) was used. Briefly, measurements included pressure pain threshold (PPT, Wagner FPK20 with 1 cm^2^ rubber tip) on painful (back) and control (trapezius) sites, temporal summation (TS, 0 g Neuropen Neurotip, Owen Mumford, Oxfordshire, UK) on painful and control sites, and conditioned pain modulation (CPM, Cole-Parmer Polystat Standard 3–6l Heat/Cool Bath). Additionally, we measured thermal (heat pain) thresholds (Medoc TSA2, Ramat-Yishai, Israel) to determine individual temperatures for the pain anticipation task in the MRI scanner (see below) (see Supplementary Material for details on each QST measure).

### Mind your pain (MyP)

Intervention details are reported elsewhere (42). Briefly, the MyP intervention involves a 1-hour virtual educational session, an illustrated handout, and a 1–2-min attention task on a smartphone app, performed several times daily, during peak pain moments for each participant, over 8 weeks. Participants receive app notifications and are encouraged to use it during peak pain moments. The task incorporates ecological momentary assessment of pain intensity and interference on a 0–10 numerical rating scale. Guided by a male voice, participants attend to specific aspects of their pain sensation, including feeling tone, motion, temperature, density, and clarity of borders. Unlike traditional mindfulness, that focuses on being aware of one's thoughts, feelings, and surroundings in the present moment without judgment, MyP focuses on detailed exploration of the pain sensation itself (72). (See Supplementary Material for details).

### Statistical analysis

Repeated measures ANOVA with group (MyP+, MyP−) as a between-subjects factor and time (Pre-tx, Post-tx) as a repeated measure was used to explore treatment effects between the two responder groups on all patient reported outcomes. Ordinal variables were examined with chi-square tests. Correction for multiple comparisons was at *p* < 0.007 for self-reported scales and *p* < 0.013 for QST measures. All *post hoc* analyses were conducted in JASP (Version 0.16.4; JASP Team, 2023).

## Results

### Demographic and clinical variables

Participants details are shown in Table 1. Subjects were 80% women, 70% non-Hispanic, White. On average, subjects experienced chronic back pain of moderate intensity (4.72 ± 1.75). There were no significant demographic differences between responders (MyP+) and non-responders (MyP−) in our study.

**Table 1:** Sample demographics.

	Full		MYP−		MYP+		Stats	
	Mean	SD	Mean	SD	Mean	SD	t/chi	pval
Age	48.6	12.2	44.14	11.24	52.67	11.94	−1.98	0.06
Sex	6M, 23F	2M, 12F	4M, 11F	0.68	0.4			
Ethnicity	No	%	No	%	No	%	6.4	0.09
Hispanic or Latino	4	14	4	29	0	0		
Not Hispanic or Latino	21	72	9	64	12	80		
Unknown	1	4	0	0	1	7		
Decline to answer	3	11	1	7	2	13		
Race	No	%	No	%	No	%	5.17	0.40
American Indian or Alaska Native Asian	1	3	1	7	0	0		
Asian	4	14	2	14	2	13		
Black or African American	1	3	0	0	1	7		
White	20	69	9	64	11	73		
Unknown	1	3	0	0	1	7		
Decline to answer	2	7	2	14	0	0		
Education	No	%	No	%	No	%	2.87	0.41
High school	1	3	0	0	1	7		
Associate or technical degree	4	15	3	21	1	7		
College or Bac. Degree	13	45	7	50	6	40		
Doctoral of postgraduate	11	38	4	29	7	46		
Employment	No	%	No	%	No	%	0.37	0.83
Full-time	15	52	8	57	7	47		
Not-employed	12	41	5	36	7	47		
Part-time	2	7	1	7	1	7		
Relationship^a^	No	%	No	%	No	%	0.76	0.86
Divorced	2	7	1	8	1	7		
Married	15	52	7	54	8	53		
Never married	8	28	3	23	5	33		
Domestic partner	1	10	0	15	1	7		
Height	66	4.3	65.7	4.9	66.33	3.74	−0.38	0.70
Weight	166	44.1	163.4	55.04	169	32.46	−0.33	0.70
Pain duration	4.48	0.63	4.57	0.51	4.4	0.74	0.70	0.47
Low back pain intensity	4.72	1.75	4.286	1.939	5.133	1.506	−1.30	0.20

“MyP+” responder [at least 50% decrease in Pain, Enjoyment of Life and General Activity Scale (PEG) (47)], “MyP−” - non-responder, <50% decreased in PEG.

^a^ *n* = 26.

### Primary outcome measure

**Figure 1:**
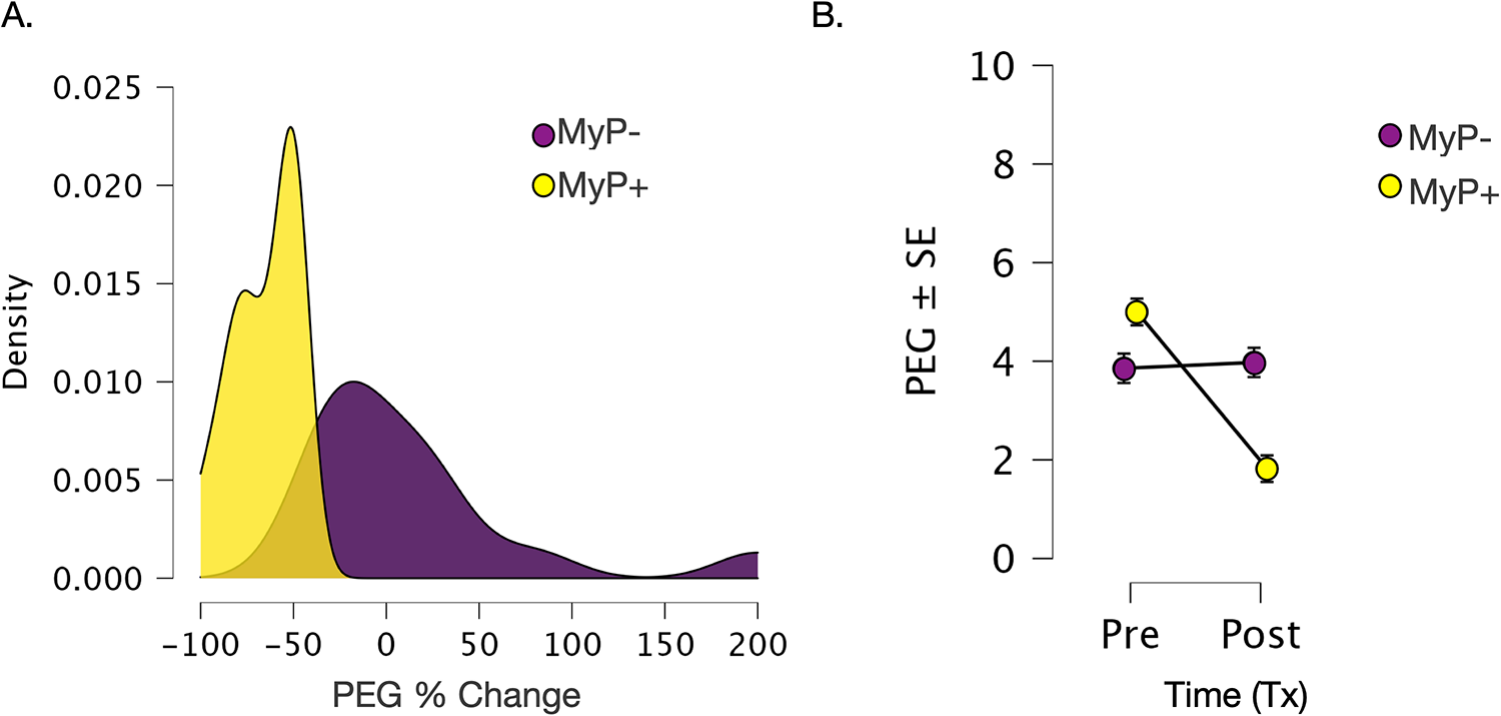
Changes in primary outcome measure (PEG). **(A)** Changes in primary outcome measure (PEG). **(A)** Density plots for %change in PEG scores over 8 weeks of treatment for responders (MyP+, yellow) and non-responders (MyP−, purple). Analysis of treatment response by responder status showed an average decrease of 65% ± 4.6% in PEG scores in the MyP+ group (yellow) and an average increase of 9.3% ± 16.8% in PEG scores in the MyP− group (purple). **(B)** Visualization of raw PEG scores overtime by the responder group. Importantly, no significant group effects were observed [F(1,27) = 3.702, *p* = 0.363], i.e., the two groups did not differ in their baseline PEG scores. As expected due to responder group definition, the effects of treatment time (PreTx, PostTx) [F(1,1, 27) = 29.24, *p* < 0.001] and time by group (MyP−, MyP+) interaction effects [F(1,1,27) = 33.964, *p* < 0.0001] were highly significant. PEG - Pain, Enjoyment of Life and General Activity Scale (PEG) (48), Tx, treatment. C.f. text for further details.

Average PEG scores decreased significantly from 4.4 ± 0.4 to 2.9 ± 0.3 (mean ± SE), which was highly statistically significant (*p* < 0.001). Analysis of treatment response by responder status showed an average decrease of 65% ± 4.6% in PEG scores in the MyP+ group and an average increase of 9.3% ± 16.8% in PEG scores in the MyP− group (visualized in Figure 1). The two groups did not differ in their baseline PEG scores [F(1,27) = 3.702, *p* = 0.363]. The effects of time (Pre-tx, Post-tx) [F(1,1, 27) = 29.24, *p* < 0.001] and time-by-group (MyP−, MyP+) interaction effects [F(1,1,27) = 33.964, *p* < 0.0001] were highly significant.

### Brain activation: task effects

**Figure 2:**
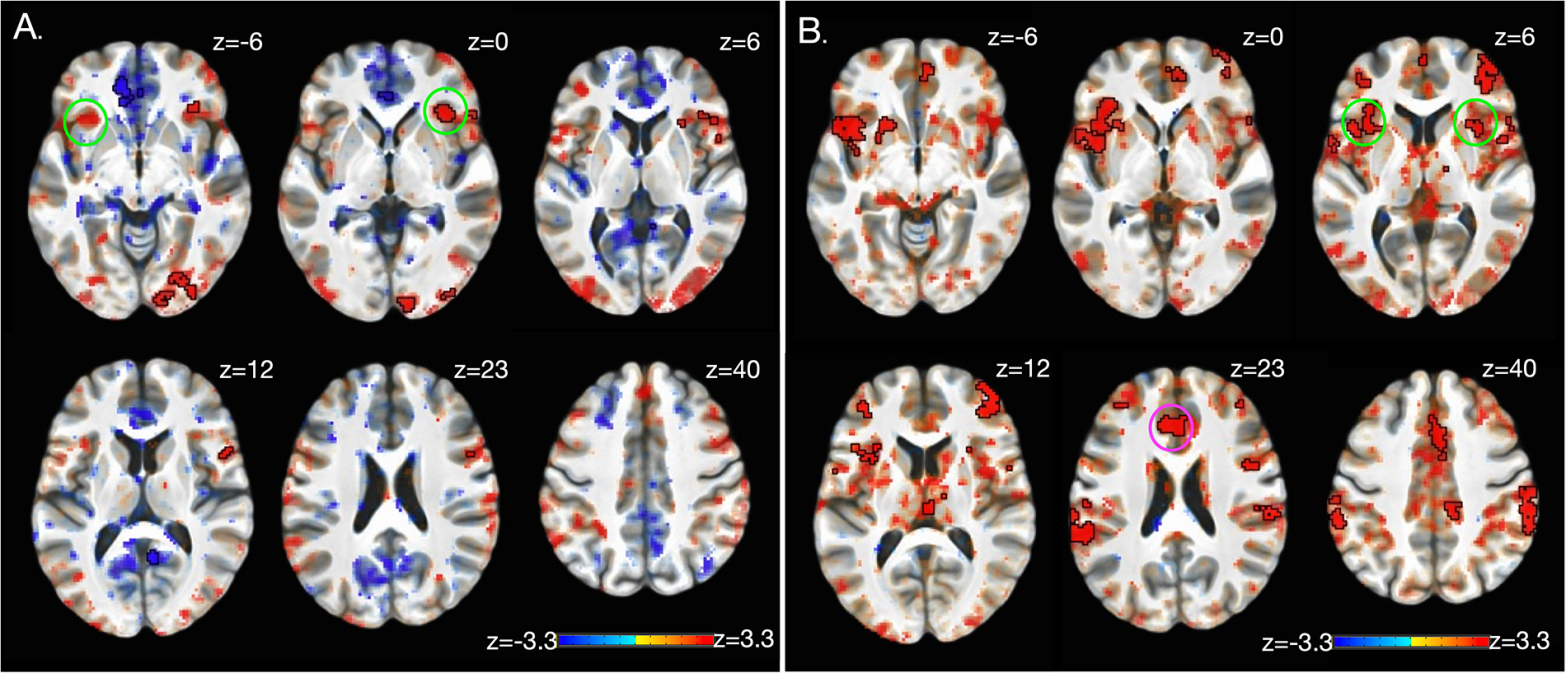
Brain activation: task effects. Significant BOLD response during high pain (HP) versus low pain (LP) anticipation **(A)** and stimulation **(B)** at baseline (i.e., before treatment) using a whole-brain approach (see Methods). The expected activation pattern was observed for both anticipation **(A)** and stimulation **(B)** periods including activation in the anterior insula (green circle), and anterior cingulate cortices (pink circle). z-stats are overlaid on the group average of the cohort's normalized anatomy. Black outlines clusters that survived significance (71), else sub-significant voxels are shown. c.f. Tables 2, 3 and text for further details. Left, left.

To ensure the desired task effects, linear contrasts were compared between the high-pain and low-pain eliciting temperatures for anticipation (Figure 2A, also Table 2) and stimulation (Figure 2B, also Table 3) periods. The expected activation pattern was observed for both anticipation and stimulation periods including activation in the anterior insula, deactivation within ventromedial PFC and PCC during pain anticipation (Figure 2A, Table 2), and activation within insula, anterior cingulate cortices, and the thalamus during painful stimulation (Figure 2B, Table 3).

**Table 2:** Anticipation.

	Baseline task effects (High-pain vs. Low-pain at baseline)				
	vol	x	y	z	Z stat
Brain region					
Right lingual gyrus	301	26	−84	−9	3.214
Right insula (RAI)	169	39	26	−1	3.260
Left mid orbital gyrus (vmPFC)	106	−9	42	−6	−3.187
Right inferior frontal gyrus	72	53	12	14	3.239
Right superior parietal lobule	64	26	−65	61	3.085
Right posterior cingulate	49	8	−53	12	−3.408
	Group by time interaction				
	vol	x	y	z	Chi stat
Brain region					
Left lingual gyrus	309	−17	−85	−5	13.722
Right lingual gyrus	159	26	−60	−8	13.601
Left middle occipital gyrus	110	−25	−85	20	12.657
Right inferior frontal gyrus	85	44	26	22	13.114
Right lingual gyrus	54	11	−57	4	12.763
Right superior occipital gyrus	53	26	−89	28	12.348
	Baseline group effects (MyP+ vs. MyP− at baseline)				
	vol	x	y	z	Z stat
Brain region					
Left lingual gyrus	72	−22	−74	−6	−3.203
Left superior occipital gyrus	53	−10	−95	5	−3.113
Left middle occipital gyrus	48	−31	−88	23	−3.228
Right fusiform gyrus	44	29	−53	−9	−3.182

**Table 3:** Stimulation.

	Baseline task effects (High-pain vs. Low-pain at baseline)				
	vol	x	y	z	Z stat
Brain region					
Left insula	620	−40	16	−1	3.280
Right supramarginal gyrus	412	61	−32	40	3.289
Left anterior cingulate cortex	373	1	23	33	3.203
Left supramarginal gyrus	362	−62	−33	29	3.234
Right middle frontal gyrus	361	41	50	11	3.404
Right inferior frontal gyrus (dlPFC)	125	52	10	15	3.309
Right poster cingulate cortex	96	11	−34	45	3.083
Right cerebellum-cortex	82	12	−68	−49	3.183
Right supramarginal gyrus	79	58	−22	20	3.252
Left inferior frontal gyrus (dlPFC)	78	−38	44	12	3.226
Right rostral cingulate	73	6	51	−3	3.297
Right superior frontal gyrus	70	28	−1	65	3.141
Right insula	42	34	16	5	3.311
Thalamus	41	5	−18	10	3.235
	Group by time interaction				
	vol	x	y	z	Chi stat
Brain region					
Right superior occipital gyrus	1,142	22	−78	29	13.513
Left middle occipital gyrus	695	−33	−89	15	13.697
Cerebellar vermis	420	−2	−50	3	13.708
Left lingual gyrus	390	−17	−75	−11	13.069
Right fusiform gyrus	214	29	−37	−15	16.007
Right posterior cingulate cortex	164	3	−39	45	14.116
Right inferior frontal gyrus	132	45	14	38	12.433
Right lingual gyrus	127	26	−62	−9	13.638
Left calcarine gyrus	105	−4	−97	2	14.328
Left Insula (vAI)	57	−43	6	−17	13.780
Left anterior cingulate (vACC)	56	1	27	−8	12.949
Right cerebellum	50	10	−44	−3	15.085
Left ventral striatum (NAc)	47	−20	5	−12	12.884
Right insula (vAI)	44	46	6	−9	13.487
Right insula (PostINS)	43	40	−15	0	13.766
	Baseline group effects (MyP+ VS. MyP− at baseline)				
	vol	x	y	z	Z stat
Brain region					
Left middle occipital gyrus	67	−22	−98	5	−3.208
Right middle orbital gyrus	52	32	56	−2	3.161
Right precentral gyrus	52	27	−14	69	3.203
Right fusiform gyrus	42	27	−38	−17	−3.308

### Brain activation: group by time

**Figure 3:**
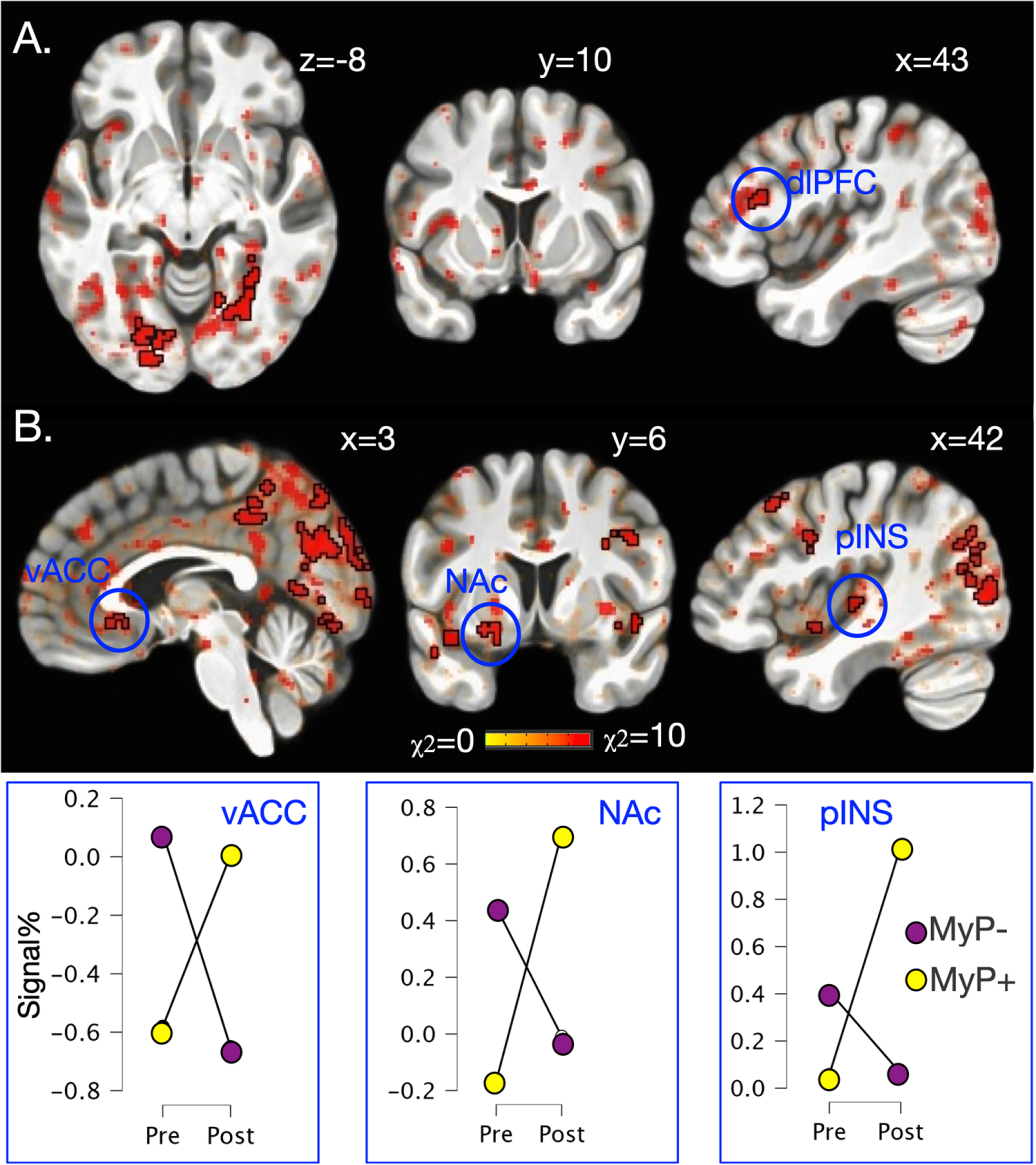
Brain activation: group by time interaction. Significant group by time interaction effects for anticipation **(A)** and stimulation **(B)** periods as calculated from the linear mixed effects model with AFNI function 3dlMEr. (69), using a whole-brain approach (see Methods). **(A)** During pain anticipation, significant group by time interaction was observed within the right prefrontal cortex (dorsolateral region, dlPFC) and several visual areas. **(B)** During pain stimulation, significant group by time interaction was observed within right posterior insula (pINS), left ventral striatum (nucleus accumbens, NAc), ventral anterior cingulate (vACC), the right prefrontal cortex (dorsolateral region), posterior cingulate and several visual areas. Bottom row plots group by time interactions during pain stimulation within vACC, NAc and pINS demonstrating that pain-related activation increased in the responder (MyP+) group while it decreased in the non-responder (MyP−) group over treatment (Tx: pre/post). Note that similar directionality was observed during anticipation (not plotted). Black outlines clusters that survived significance (71), else sub-significant voxels are shown. c.f. Tables 2, 3 and text for further details. Left, left.

Significant whole brain interaction effects are shown in Figure 3A (and Table 2) for anticipation (high and low pain anticipation) and Figure 3B (and Table 3) for stimulation (high and low pain stimulation) periods. During pain anticipation, a significant group-by-time interaction was observed within the right prefrontal cortex (dorsolateral region) and several visual areas. During pain-related stimulation, a significant group-by-time interaction was observed within the bilateral ventral anterior insulas, right posterior insula, left ventral striatum (nucleus accumbens), ventral anterior cingulate, the right prefrontal cortex (dorsolateral region), posterior cingulate and several visual areas. Examination of this interaction showed that both, anticipation- and pain-related activation increased in the MyP+ group while it decreased in the MyP− group over treatment (Figure 3).

### Brain activation: group effects

To examine baseline group differences, linear contrasts were compared between the two groups at baseline for anticipation (high and low pain anticipation) (Table 2) and stimulation (high and low pain stimulation) (Table 3) periods. Only a few regions showed significant between-group differences in the whole brain at baseline. All were located in the visual occipital regions (see Tables 2, 3).

### Baseline self-reported outcome measures

Groups were examined on several pain coping scales and QST measures at baseline, i.e., before starting the intervention. No significant baseline between group differences were observed in any of the examined measures after correcting for multiple comparisons (Table 4).

**Table 4:** Self-report and sensory measures.

	Full		MyP−		MYP+		Stats	
	Mean	SD	Mean	SD	Mean	SD	*t*	*p*
Psychological variables								
Pain anxiety symptoms (PASS20)	13.07	8.84	12.5	9.3	13.6	8.7	−0.33	0.70
Pain catastrophizing (PCS)	8.52	5.80	9.29	6.8	7.80	4.5	0.68	0.50
Perceived stress (PSS4)	7.56	1.59	8.00	1.79	7.13	1.3	1.49	0.20
Fear avoidance belief (FABQ)	11.97	6.57	14.28	6.35	9.80	6.2	1.92	0.07
Chronic pain acceptance (CPAQ)	29.97	7.02	29.42	7.34	30.47	6.9	−0.39	0.70
Pain self-efficacy (PSEQ)	17.25	5.19	16.62	5.07	17.80	5.4	−0.6	0.60
Interoceptive awareness (MAIA2)	2.72	0.45	2.71	0.37	2.72	0.5	−0.06	0.95
Five facets of mindfulness (FFMQ)	70.21	4.98	71.00	4.80	69.50	5.2	0.77	0.45
Pain relief expectation	2.67	2.79	2.75	2.5	2.60	3.1	0.14	0.89
Patient global improvement (PGIC)	3.04	0.88	3.15	0.98	2.93	0.8	0.65	0.52
Quantitative sensory testing (QST)								
Pressure pain threshold (PPT)^a^	0.76	2.13	0.30	2.60	1.20	1.5	−1.13	0.30
Temporal summation (TS)^b^	−0.02	0.85	−0.20	0.95	0.15	0.7	−1.18	0.30
Conditioned pain modulation (CPM)^c^	0.11	1.40	−0.28	1.28	0.45	1.4	−1.40	0.20
Thermal pain threshold (°C)	44.55	1.18	44.1	1.14	45	1.1	2.26	0.03*

^a^ kgf/cm^2^, positive = higher PPT on the back pain compared to control site (c.f. text for details).

^b^ Numerical Rating Scale (1–10), difference in rating between back pain and control site.

^c^ Positive = greater CPM.

* Significant findings are denoted with an asterisk.

### Self-reported outcome measures over treatment

**Figure 4:**
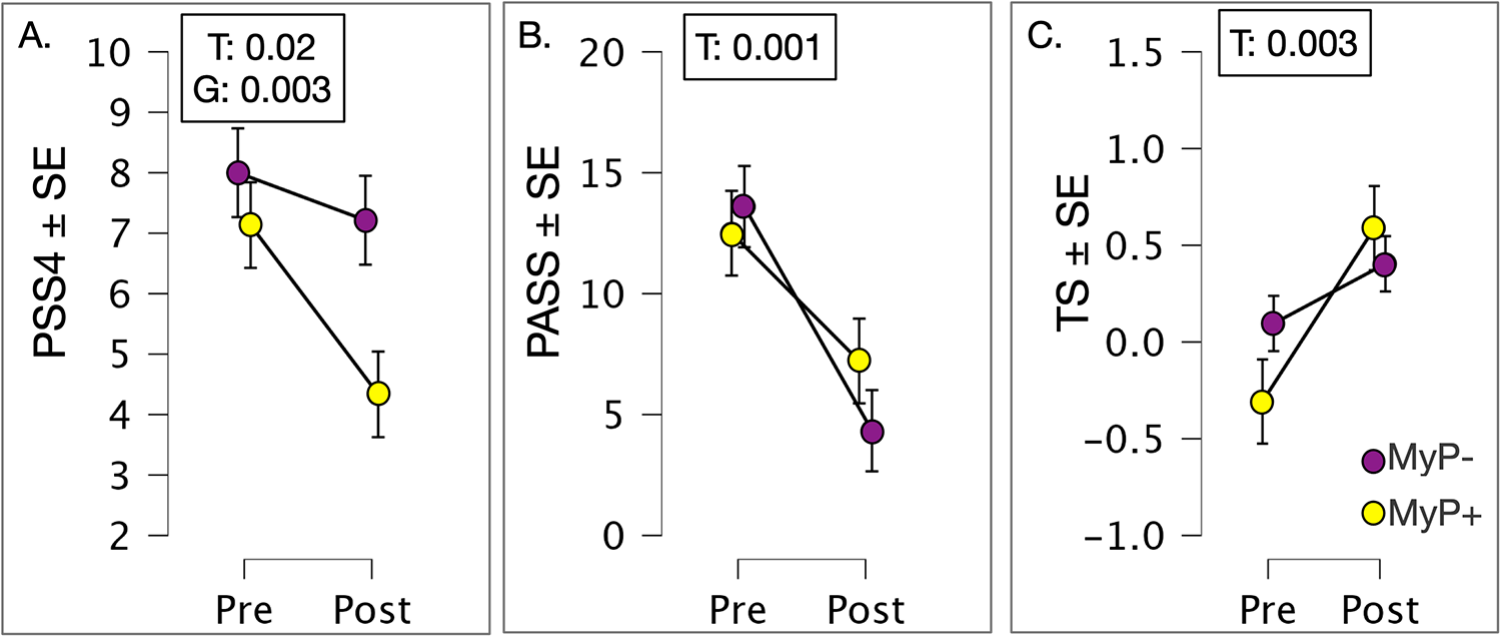
Patient reported outcomes. **(A)** Perceived stress scale (PSS4) showed significant time (F(1,1,27) = 6.180, *p* = 0.019 (approaching correction for multiple comparisons) and group effects [F(1,27) = 10.723, *p* = 0.003], with non-responders (MyP−) rating higher on this scale and showing smaller decreases with treatment. **(B)** Pain anxiety symptoms scale (PASS20) showed significant time effects [F(1,1,28) = 18.004, *p* < 0.001], demonstrating substantial decreases in both groups. **(C)** QST measure of the temporal summation of pain (i.e., higher temporal summation on the site of chronic pain compared to the control site) showed significant time effects [F(1,2,25) = 11.000, *p* = 0.003], whereby more temporal summation was observed on pain site compared to control site overtime. MyP+, treatment responders; MyP−, treatment non-responders; T, time effects; G, group effects; GxT, group by time effects. Significance *p* levels are shown. c.f. text for further details.

We explored changes in self-reported outcomes with repeated measures ANOVA (Figure 4). The perceived stress scale (PSS4) showed significant time [F(1,1,27) = 6.180, *p* = 0.019] (at trend level once corrected for multiple comparisons) and group effects [F(1,27) = 10.723, *p* = 0.003], with non-responders (MyP−) rating higher on this scale and showing smaller decreases with treatment. Likewise, the pain anxiety symptoms scale (PASS20) showed significant time effects [F(1,1,28) = 18.004, *p* < 0.001], demonstrating substantial decreases in both groups. Among QST measures examined, only the temporal summation of pain (i.e., higher temporal summation on the site of chronic pain compared to the control site) showed significant time effects [F(1,2,25) = 11.000, *p* = 0.003], whereby more temporal summation was observed on the pain site compared to control site overtime.

### Multidimensional assessment of interoceptive awareness scales (MAIA-2)

**Figure 5:**
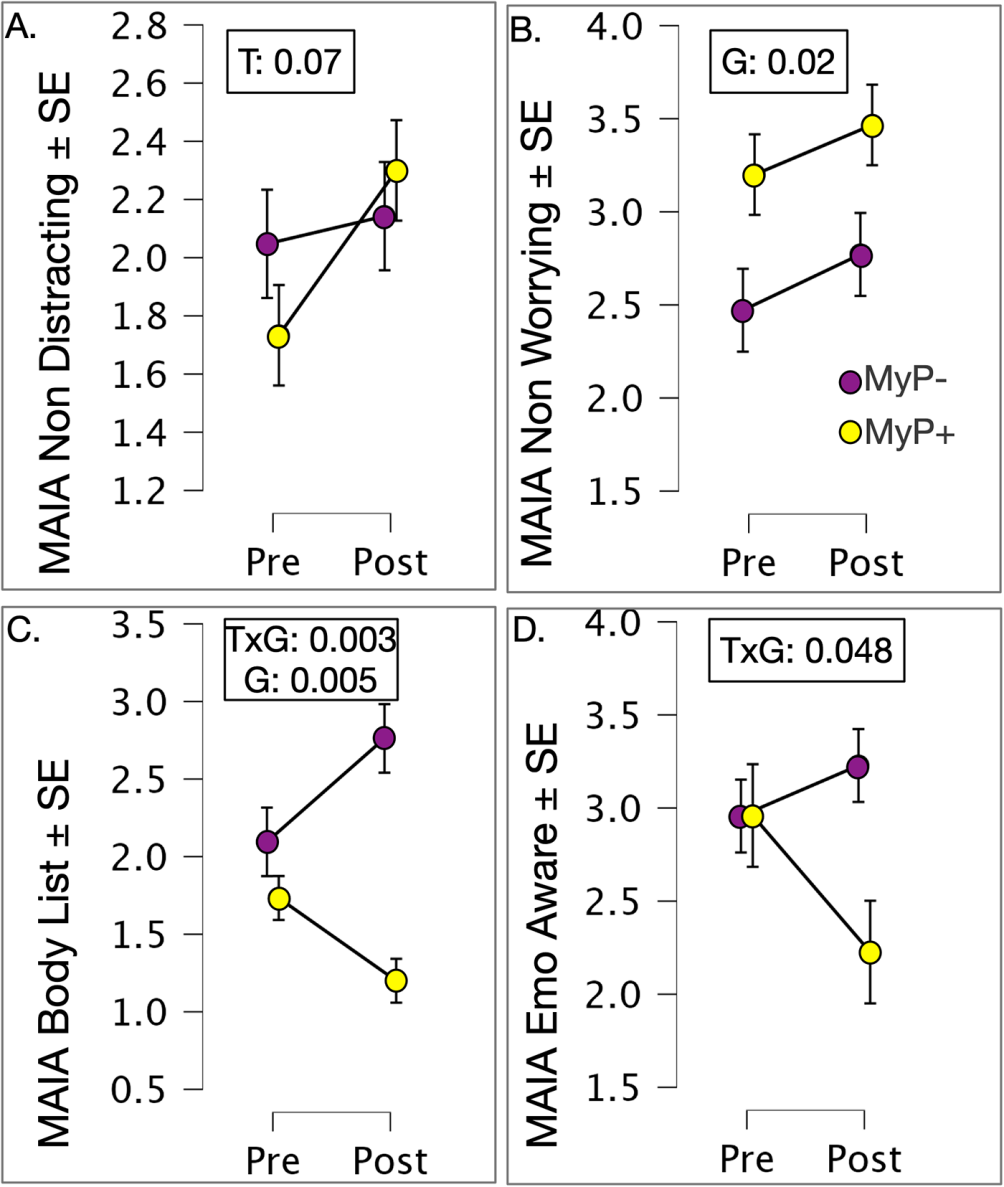
Exploration of MAIA-2 subscales by treatment response. **(A)** Time effects on MAIA Non-distracting subscale approached significance [F(1,2,27) = 3.407, *p* = 0.076]; **(B)** Significant group effects were observed for MAIA Non-worrying subscale [F(1,27) = 6.378, *p* = 0.018]. **(C)** Significant group by time interactions for MAIA-2 Body Listening [F(1,1,27) = 10.755, *p* = 0.003], and **(D)** Significant group by time interactions for Emotional Awareness [F(1,1,27) = 4.304, *p* = 0.048] subscales. Closer examination of these interactions showed that rating on these scales decreased with treatment in the MyP+ group while it increased or (remained unchanged) in the MyP− group. *p*-values are shown for significant effects of group (**G**), time (**T**) or group by time (GxT) interactions.

As mentioned above, we used low total scores on MAIA-2 to pre-select individuals for MyP intervention with the hypothesis that MAIA scores would improve, especially in relation to Non-Distraction. Since MAIA was *a priori*, we examined changes in MAIA scales without correcting for multiple comparisons (Figure 5). Significant effects from the repeated measures ANOVA are shown in Figure 5. Time effects on MAIA Non-distracting subscale approached significance [F(1,2,27) = 3.407, *p* = 0.076, Figure 5A]. Significant group effects were observed for MAIA Non-worrying subscale [F(1,27) = 6.378, *p* = 0.018, Figure 5B]. Of note are significant group-by-time interactions for MAIA-2 Body Listening [F(1,1,27) = 10.755, *p* = 0.003, Figure 5C] and Emotional Awareness [F(1,1,27) = 4.304, *p* = 0.048, Figure 5D] scales. Closer examination of these interactions showed that rating on these scales decreased with treatment in the MyP+ group while it increased or (remained unchanged) in the MyP− group.

### Exploring neural differences in PEG by treatment response at baseline

**Figure 6:**
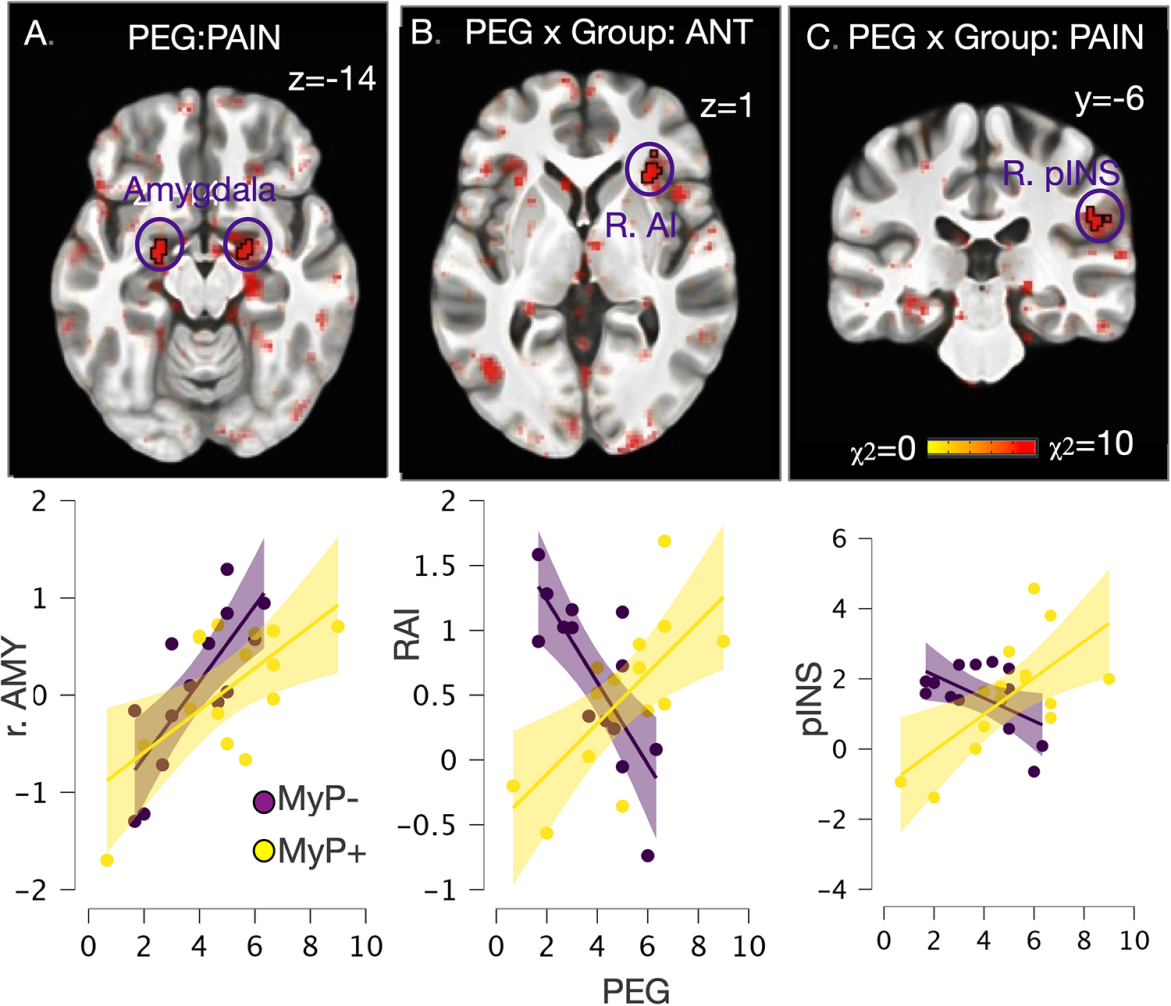
Whole brain exploration of the interaction between endogenous (as measured by PEG) and experimental pain at baseline. **(A)** Significant main effects of PEG at baseline (i.e., in both responder groups before treatment) were noted within bilateral amygdala during experimental pain stimulation (left: −21/−5/−13, 28 voxels, χ^2^ = 14.6, *p* < 0.001; right: 20/−5/−13, 28 voxels, χ^2^ = 14.6, *p* < 0.001), while no regions survived significance during the anticipation period; **(B)** Significant PEG by Group interaction was observed within right anterior insula (AI) (31/27/2, 36 voxels, χ^2^ = 10.7, *p* < 0.005) during pain anticipation; **(C)** Significant PEG by Group interaction was observed within right posterior insula/parietal operculum (52/−28/26, 33 voxels, χ^2^ = 10.6, *p* < 0.005) during pain stimulation. Scatter plot detail these interactions, which showed that in the MyP+ group those with higher PEG scores at baseline also showed higher right AI activation during anticipation and right pINS/parietal operculum activation during painful stimulation. The opposite relationship was noted in the MyP− group. AMY, amygdala; RAI, right anterior insula; pINS, posterior insula; PEG, pain, enjoyment of life and general activity scale.

The results of linear mixed effects models conducted on baseline brain response, i.e., before treatment, with PEG as a factor are shown in Figure 6. Note that PEG scores did not differ between the two responder groups at baseline (see Figure 1). Main effects of PEG were noted within bilateral amygdala (Figure 6A) during experimental pain stimulation (left: −21/−5/−13, 28 voxels, χ^2^ = 14.6, *p* < 0.001; right: 20/−5/−13, 28 voxels, χ^2^ = 14.6, *p* < 0.001), while no regions survived significance during the anticipation period. Conversely, significant PEG-by-group interaction was observed within right anterior insula (AI) (31/27/2, 36 voxels, χ^2^ = 10.7, *p* < 0.005) during pain anticipation (Figure 6B) and right posterior insula/parietal operculum (52/−28/26, 33 voxels, χ^2^ = 10.6, *p* < 0.005) during pain stimulation (Figure 6C). Examination of these interactions showed that in the MyP+ group, those with higher PEG scores at baseline also showed higher right AI activation during anticipation and right pINS/parietal operculum activation during painful stimulation. The opposite relationship was noted in the MyP− group (Figures 6B,C).

## Discussion

The goal of this study was to assess whether a mobile app-based interoceptive attention intervention in individuals suffering from cLBP results in treatment response-related changes within interoceptive neurocircuitry during an experimental pain task, whereby treatment response is defined by at least 50% reduction in PEG scores (49). Our major findings are as follows: *First*, treatment response was associated with increased activation in the contralateral posterior insula and bilateral ventral anterior insula in the task-based fMRI during stimulation with experimental pain. *Second*, additional increases in experimental pain-related brain activation were noted in the ventral anterior cingulate, dorsolateral prefrontal cortex, and nucleus accumbens. *Third*, although treatment responders did not differ at baseline from non-responders in experimental pain-related interoceptive brain activities, we observed baseline differences in the associations between their endogenous back pain PEG scores with their brain activity in the anterior insula and posterior insula/parietal operculum.

Regarding secondary treatment outcomes, and contradicting our a-priori hypothesis, we did not observe significant improvements in overall self-reported interoceptive awareness (MAIA-2) for our entire study sample over time, except for a trend decrease in distraction, as measured by MAIA-2 Non-Distraction. Self-reported perceived stress and pain anxiety, as well as QST temporal summation of pain, significantly improved irrespective of treatment response. Yet, when looking at treatment responders compared to non-responders, MAIA-2 dimensions of Body Listening and Emotional Awareness significantly improved in responders.

In summary, this work suggests that the MyP intervention may effectively improve cLBP (by PEG scores for pain intensity and interference) and alter experimental pain-related brain activation. Due to notable baseline differences in the association between chronic pain PEG scores with brain activity in the insula during experimental pain stimulation, we posit that our innovative intervention may be most successful in individuals with task-based insula activation response to acute experimental pain.

Specifically, as hypothesized, we found increased brain activation within the contralateral posterior insula, a key region in the interoceptive sensory cortex (12), and bilateral ventral anterior insula in those with cLBP who responded to the intervention (MyP+). Our *a priori* hypotheses were based on the clinical observations that patients with cLBP often prefer distraction and avoidance over interoceptive awareness through mindful attention to their pain (33). This distraction from, or inattention to, pain can manifest as decreased insula responses to experimental pain (35–37). Thus, our findings provide initial evidence that a successful response to an interoceptive intervention that trains pain-focused attention and overcomes a habitual pain coping style of distraction is associated with functional brain changes related to experimental pain within the interoceptive network. These results align with recent neuroimaging studies showing plasticity within the insula and interoceptive neurocircuitry following a Mindful Awareness in Body-oriented Therapy intervention in healthy volunteers (40). However, we cannot infer with confidence whether the observed experimental pain-related brain changes were a result of a mechanistic (aka plasticity) or a predictive (based on *a priori*) nature (51). In other words, it is plausible that the observed changes in experimental pain-related brain activation may have been caused by treatment, secondary factors (e.g., being in a “correct head space” to respond to treatment), or a combination of the two. Further data on experiences of treatment and treatment engagement and future RCT could help better explain the underlying mechanism. Nevertheless, our findings may provide further evidence that changes within interoceptive circuitry including insula and anterior cingulate, which together serve as the homeostatic/emotional/limbic sensorimotor cortex (26) and provide adaptive control of the body and its autonomic functions (27), may serve as an objective biomarker of one of the important effects of mind-body approaches that is relevant for reducing pain in cLBP.

We also observed treatment-related changes in experimental pain-induced brain response within the ventral anterior cingulate, dorsolateral prefrontal cortex, and nucleus accumbens. The dorsolateral prefrontal cortex is implicated in cognitive pain modulation (50). Likewise, human studies show that the offset of pain (or pain relief) is associated with positive activity change in the rostral and dorsal parts of the anterior cingulate and ventral striatum in humans (73, 74), circuitry that is implicated in pain relief mechanisms in animals (75). Thus, the observed treatment response-related changes within these regions in our study may suggest improved and/or reconditioning of the adaptive response to both experimental and endogenous cLBP.

Even though the two responder groups did not exhibit substantial neural differences in experimental pain-related interoceptive circuitry at baseline, there were significant distinctions related to the impact of their endogenous back pain. Notably, these differences were observed despite the absence of significant variations in behavioral, PEG (Pain, Enjoyment, and General Activity) scores, or Quantitative Sensory Testing (QST) measures between responders and non-responders at baseline. Specifically, we found a significant positive association between the impact of the endogenous back pain (PEG) and increased right AI activation during experimental pain anticipation and right pINS/parietal operculum during experimental pain stimulation in the responder group at baseline, consistent with an adaptive, non-avoidant way to react to pain (35–37, 50). The opposite was true in the non-responder group, despite similar ratings of both the endogenous pain impact (PEG) and experimental heat pain intensity in the MRI scanner. Although no significant baseline differences in fear-avoidance beliefs were observed (a tendency was noted), we can speculate that treatment-related brain changes in pain anticipation and processing in responders were facilitated by less avoidance/distraction at baseline, as suggested by their neural response.

Although total MAIA-2 scores did not change significantly with treatment, significant group-by-time interactions were observed for MAIA-2 Body Listening and Emotional Awareness scales, which decreased with treatment in the MyP+ group, whereas they increased (or remained unchanged) in the MyP− group. A hypothesis for the mechanism for this result may be that responders could have realized that they overestimated their skills before the intervention and became more aware of their skill limits. Interestingly, non-responders (MyP−) rated higher on the perceived stress scale at both time points (consistent with lower scores on the MAIA-2 Non-Worrying scale), which showed some improvement over time. We also found that pain anxiety decreased over time irrespective of treatment response. This may suggest that MyP reduces perceived stress, chronic pain-related anxiety, and the threat value of pain (76) by refocusing the patients' attention on interoceptive attributes.

Furthermore, the intended altered perception of pain by MyP via practicing an interoceptive attention focus is further supported by the observed effects on the temporal summation of pain. We found that irrespective of MyP response, subjects showed increased temporal summation of pain post-treatment compared to pre-treatment, whereby repeated application of painful stimuli to the back pain site was perceived as more subjectively painful over the course of treatment. Temporal summation reflects the progressive increase in dorsal horn neuronal firing in response to repetitive C-fiber stimulation (i.e., CNS sensitization), common in chronic pain patients (77, 78). It is plausible that our patients learned how to experience their endogenous back pain, as instructed, with more awareness rather than using distracting thoughts. However, we believe that responders (MyP+) could generalize those changed thoughts and interoceptive experiences to experimental pain and benefit from them when experimental pain was applied, reflected in their brain activation, while non-responders (MyP−) did not. Alternatively, it is also possible that changes in temporal summation were related to sensory habituation, although we did not observe habituation to temperature stimuli in our sample. Nevertheless, future randomized controlled studies may address this interpretation.

This study has some important limitations. Primarily, our study had a single-arm design and lacked a control group. Nevertheless, this study is the first to assess the preliminary efficacy, brain mechanisms, and behavioral effects of a novel interoception-based mobile intervention. This pioneering exploration sets the stage for further randomized controlled trials (RCT) and establishes a proof of concept for the intervention's potential. This was an open-label treatment study where we dichotomized our patients based on responder status. Although our study design limited mechanistic interpretation due to the absence of a control group, the dichotomized treatment outcomes offer valuable insights into the direction and extent of successful treatment, generating robust hypotheses for future investigations. In addition, our cohort was primarily female and white. Although it lines up well with the global chronic pain demographics, the translational value of our intervention may be limited and needs further investigation. Although our treatment responder groups did not significantly vary in age or ethnicity, future larger studies need to examine the effect of age and ethnicity on the utility of similar interventions. Another limitation is that the expected improvement in overall interoceptive awareness and mindfulness was not found. This finding indicates a potential sequential relationship, suggesting that changes in attention and distraction precede shifts in awareness and mindfulness. This is in line with findings in patients with cLBP and co-morbid depression that showed a sequential mediation of attention decentering *before* self-reported interoceptive awareness for mediating the effects of a mindfulness intervention (79).

Future research can explore these dynamics in extended follow-up evaluations within the clinical treatment arc and RCT designs.

To summarize, the current study introduces a novel mobile intervention that, subject to further validation through randomized controlled trials, represents a potential paradigm shift in pragmatic chronic pain management. Unlike conventional methodologies that primarily advocate for pain diversion, our intervention aims to cultivate a mindful, neutral, and non-evaluative mode of attention. This approach focuses on promptly perceiving pain rather than engaging in cognitive processes like rumination or worry. Furthermore, the outcomes of our investigation provide insights into the underlying cortical mechanisms of non-opioid, interoception-focused therapies for individuals suffering from cLBP. This work is poised to lay the foundation for a novel future path in pain management interventions.

## Data Availability

The raw data supporting the conclusions of this article will be made available by the authors, without undue reservation.
