# Supplementary material for: Enhancing chronic low back pain management: an initial neuroimaging study of a mobile interoceptive attention training
Source: Front Pain Res (Lausanne). 2024 Sep 30;5:1408027. doi: 10.3389/fpain.2024.1408027 (PMC11471628; doi:10.3389/fpain.2024.1408027)
Supplement: Supplementary file 1 [file Datasheet1.pdf]

Supplementary Material

Supplementary Data

Participants: Thirty participants (6 males, mean ± SD age: 49±12, range 26-64) gave written informed consent to participate in this study, which was approved by the UCSF Human Research Protection Program and San Francisco Veterans Affairs Healthcare System Research and Development Committee (SFVAHS RDC), and registered with ClinicalTrials.gov #NCT06186193. All methods were performed in accordance with the relevant guidelines and regulations. Participants were recruited through university newsletters, research and study websites, flyers, and social media. Study inclusions were: 1) English-speaking men and women aged 18-65 years old; 2) experiencing chronic back pain (cLBP) defined according to the NIH Research Task Force Recommendation on Research Standards for cLBP [1-3] (i.e., pain at least half the days in the past 6 months, at least 3 out of 10 intensity on the numeric rating scale (NRS)); 3) own a smart phone; and 4) demonstrating low levels of interoceptive awareness and habitual distraction from the Multidimensional Assessment of Interoceptive Awareness scales (MAIA-2).[4] Low level was defined as: a) a MAIA-2 summary score below the population mean score of 3.41 [range 0-5] [5] and b) a MAIA-2 Non-Distraction score below 2.91 [range 0-5]. These criteria were chosen to test our hypothesis that the mindful attention task will be able to increase below average interoceptive awareness and restore interoceptive insula activation in cLBP patients, who are used to distracting themselves from their pain experience. Subjects were excluded from the study if they: 1) had current or history of spine infection, spine tumor, vertebral fracture, cauda equina syndrome; radiculopathy or sciatic pain was not excluded if the condition was stable and did not lead to significant movement restrictions or ≤4/5 muscle strength; 2) had a current or lifetime history of bipolar disorder, psychosis, eating disorders, or obsessive compulsive disorder (OCD); 3) had a history of alcohol and/or drug dependence and/or moderate-severe abuse within 3 months of study participation; 4) showed a neurological disorder that might be associated with cognitive dysfunction (including head trauma associated with fracture, traumatic brain injury (TBI) resulting in loss or alteration of consciousness > 10 min); 5) had significant other medical conditions (malignancies, liver failure, renal failure, pain conditions from inflammatory diseases, abdominal aortic aneurysm, muscle weakness from radiculopathy); 6) met MRI-related exclusions (e.g., irremovable ferromagnetic material, including non-removable jewelry, braces, or permanent dental retainers; were pregnant; claustrophobic; colorblind and left-handed (due to the nature of the task); were unable to lie still for approximately 60 minutes; had prior neurosurgery; had older tattoos with metal dyes); 7) were involved in a lawsuit or Worker's Compensation claim related to their back; 8) received steroid or *Botox* injections near the spine in the past 3 months. One female participant was excluded from the analyses due to extensive motion in the MRI scanner. The final sample included 29 participants with cLBP (6M; age: 48.6 ± 12.2 years old).

Patient-Reported Outcomes (PROs) Ways of thinking about pain and strategies to cope with pain have an impact on patients' behavior and quality of life. **Pain catastrophizing**, or ruminating about and magnifying pain, and having a sense of helplessness, is assessed by the BMD-required Pain Catastrophizing Scale, 6-item version.[6-8] The BWG recommends that **fear of movement** be assessed by the physical activity subscale of the Fear Avoidance Beliefs Questionnaire (FABQ)[9]; however, the Tampa Scale of Kinesiophobia[10, 11] is an alternative option. A similar concept is **pain avoidance**, which can be assessed via the Pain Anxiety Symptoms Scale short form[12, 13].

Adaptive coping with pain is also important to assess. **Pain acceptance** is measured via the Chronic Pain Acceptance Questionnaire-8 (CPAQ).[14],[15] **Pain self-efficacy,** or one's belief in being able to effectively control or cope with pain, is a BWG recommended construct that can be assessed via the 4-item version of the Pain Self-Efficacy Questionnaire.[8]

Quantitative Sensory Testing (QST): A modified protocol from the German Research Network on Neuropathic Pain (DFNS [18]) was used. All QST procedures occurred prior to scanning. Three separate tests were performed at baseline and at 8 weeks (pressure pain sensitivity, conditioned pain modulation and temporal summation of pain). Pressure pain sensitivity was assessed using an analog algometer with a 1-cm2 rubber probe (FPK20, Wagner Instruments, Greenwich, CT, USA) to quantify pressure pain thresholds (PPT). The primary test site was located in the lumbar region by the participant's response to manual over-pressure (springing palpation) performed in the prone position. The control site was located over the contralateral trapezius muscle (diagonal from lumbar site). Pressure was manually increased at a rate of rise of 0.5 kgf/cm2/s (10 kg max, metronome guided) until participants first report that the pressure sensation becomes painful. Pressure intensity (in kgf/cm2) read from the algometer at that time was considered the PPT. Measurements were conducted 3x/site with 60-s rest intervals between each pressure application. Probe placement was varied slightly trial to trial to prevent sensitization from repeated testing of the same site. Mean PPT was used for further analysis.

Conditioned Pain Modulation (CPM) and Temporal Summation of Pain (TS) are considered dynamic psychophysics and are thought to depict endogenous pain modulation. Briefly, conditioned Pain Modulation (CPM) is a non-invasive experimental method to assess the integrity of descending analgesic pathways, at least in part mediated by the release of endogenous opioids. As recommended, pressure algometer and water bath (hot water immersion) were used. Temporal Summation (TS) was evaluated using the Neuropen and the established method. TS reflects the progressive increase in dorsal horn neuronal firing in response to repetitive C-fiber stimulation (i.e., CNS sensitization), common in chronic pain.

Variables of interest used in the evaluation of treatment response were: 1) pressure pain threshold (PPT) (calculated as the difference between pain and control sites thresholds, the more positive the difference the less pressure sensitivity on the patient's back pain site), 2) temporal summation (TS) (calculated as the difference between temporal summation on the pain and controls sites, the more positive the difference the more temporal summation on the patient's back pain site, and 3) conditioned pain modulation (CPM) (calculated as the difference in PPT between control site before and after application of secondary, conditioning pain stimulus (i.e., hand hot water bath immersion in this study; the more positive the difference the greater CPM)). In addition, we measured thermal (heat pain) thresholds (Medoc TSA2, Ramat-Yishai, Israel) to determine individual temperatures for the pain anticipation task in the MRI scanner.

Mind your Pain (MyP): The MyP consists of an individual 1-hour virtual educational introduction session, a 6-page illustrated handout summarizing the discussed pain education, and a 1 to 2-minute attention task subsequently performed several times per day over 8 weeks. The task is downloaded on a smart phone app with notifications twice per day (prompt times according to participant scheduling preference). In addition, participants are asked to use the app whenever they perceive their pain at its worst. The task included an ecological momentary assessment (EMA) of pain intensity and interference each answered on a 0-10 NRS followed by a brief auditory guidance into attending to pain as the sensation "where it is the most intense". A male voice guided the attention focus into the

center of “the sensation we call pain” in a detached and equanimous way and asks to carefully observe and explore it with curiosity in regards to five components of that sensation: feeling tone (sharp versus dull), motion (static versus movement/change), temperature (cold versus hot); density (tense/tight versus loose), and clarity of the borders of the perceived three-dimensional shape (diffuse versus clearly-defined). The MyP intervention is markedly different from common mindfulness interventions for pain in that it includes a very specific focus on the phenomenological details of the pain sensation itself, whereas common mindfulness interventions use a more general mindful awareness focus on the body in space, exteroceptive sensations, thoughts, and emotions.[19]

MRI Pain-Anticipation Task: Each fMRI session included two acquisitions (i.e., Run 1 and Run 2) of the pain-anticipation paradigm, separated by 5-7 mins. The stimulation was delivered through a $9cm^2$ thermode (Medoc TSA2, Ramat-Yishai, Israel) on the participant's left forearm, as described elsewhere [20]. The schedule of stimuli differed between imaging runs in a pseudorandom and counterbalanced order. The periods of anticipation were ten seconds long and began with a cue that signaled high, low, or uncertain level of pain. More specifically, ten seconds prior to the onset of pain, the participants were presented with an image of a colored cross. A red cross indicated a temperature stimulus producing moderate levels of heat pain or “high-pain, HP”, a green cross indicated a temperature stimulus producing low levels of heat pain or “low-pain, LP”, and a yellow cross, indicated pain of uncertain intensity (at 50% probability being high or low, which was not known to the subject). These anticipation periods were followed by seven seconds of either high or low-pain. The high-pain/low-pain stimulation was determined based on individual thermal pain thresholds, both of which had a rise and fall rate of 10ºC/sec. In all instances when the level of pain was cued, the participant received the corresponding level of pain. When an uncertain cue was given, the participant was administered either the HP or LP stimulation. Each temperature stimulus was followed by a period of rest, signaled by a change in the color of the cross to blue, that was jittered between 24 to 30 seconds (aside from the short period of rest before the first anticipation cue in each session, which lasted 7 and 10 seconds, respectively). Each session included 14 separate anticipation-pain conditions and lasted a total 618 seconds. In Run 1, there were three HP-cued conditions and four LP-cued conditions. The other seven conditions began with an uncertain cue (UN), three of which were followed by low-pain delivery, and four with high-pain. In Run 2, there were four HP conditions, three LP conditions, and of the seven UN conditions, four were followed with low-pain and three were followed with high-pain. In combination, there was a total of seven HP, seven LP, and fourteen UN (with seven LP and seven HP) conditions.

Supplementary Figures

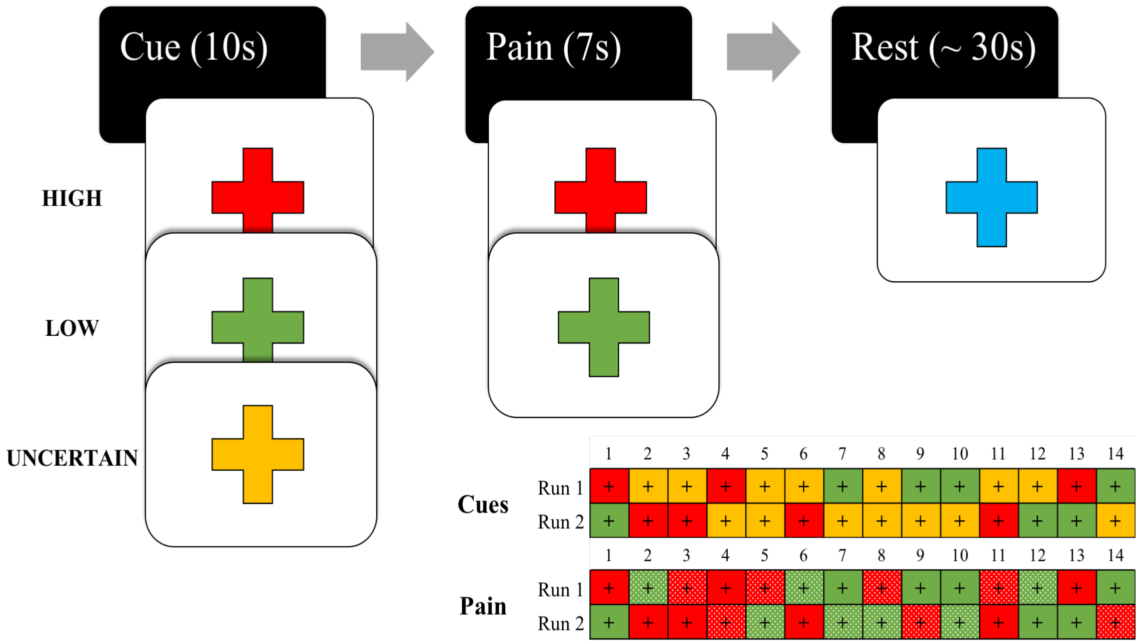

Supplementary Figure 1: Schedule of pain-anticipation paradigm. Simple breakdown of ordered pain-anticipation-rest timeline. Cues appear as presented to subjects. RED represents high-pain cue followed by high-pain administration (HP). GREEN represents low-pain cue followed by low-pain administration (LP). YELLOW represents uncertain pain cue followed by either high or low-pain administration (UN). Bottom right: timeline for one complete imaging session (both run 1 and run 2) separated by anticipation cue timeline (HP, LP, and UN), and pain stimulation (HP and LP).

References

1. Deyo, R.A., S.F. Dworkin, D. Amtmann, G. Andersson, D. Borenstein, E. Carragee, et al., *Report of the NIH task force on research standards for chronic low back pain.* Spine (Phila Pa 1976), 2014. **39**(14): p. 1128-43.
2. Protocol, A.T.I., *Trauma-informed care in behavioral health services.* Rockville, USA: Substance Abuse and Mental Health Services Administration, 2014.
3. Treede, R.D., W. Rief, A. Barke, Q. Aziz, M.I. Bennett, R. Benoliel, et al., *A classification of chronic pain for ICD-11.* Pain, 2015. **156**(6): p. 1003-1007.

4. Mehling, W.E., M. Acree, A. Stewart, J. Silas, and A. Jones, *The Multidimensional Assessment of Interoceptive Awareness, Version 2 (MAIA-2).* PLoS One, 2018. **13**(12): p. e0208034.
5. Mehling, W.E., J. Daubenmier, C.J. Price, M. Acree, E. Bartmess, and A.L. Stewart, *Self-reported interoceptive awareness in primary care patients with past or current low back pain.* J Pain Res, 2013. **6**: p. 403-18.
6. Sullivan, M.B., SR, *The Pain Catastrophizing Scale: Development and Validation.* Psychological Assessment, 1995. **7**(4): p. 524-532.
7. Osman, A., F.X. Barrios, B.A. Kopper, W. Hauptmann, J. Jones, and E. O'Neill, *Factor structure, reliability, and validity of the Pain Catastrophizing Scale.* J Behav Med, 1997. **20**(6): p. 589-605.
8. McWilliams, L.A., J. Kowal, and K.G. Wilson, *Development and evaluation of short forms of the Pain Catastrophizing Scale and the Pain Self-efficacy Questionnaire.* Eur J Pain, 2015. **19**(9): p. 1342-9.
9. Waddell, G., M. Newton, I. Henderson, D. Somerville, and C.J. Main, *A Fear-Avoidance Beliefs Questionnaire (FABQ) and the role of fear-avoidance beliefs in chronic low back pain and disability.* Pain, 1993. **52**(2): p. 157-68.
10. Hudes, K., *The Tampa Scale of Kinesiophobia and neck pain, disability and range of motion: a narrative review of the literature.* The Journal of the Canadian Chiropractic Association, 2011. **55**(3): p. 222-232.
11. Miller, R.P., S.H. Kori, and D.D. Todd, *The Tampa Scale: a Measure of Kinisophobia.* The Clinical Journal of Pain, 1991. **7**(1).
12. McCracken, L.M., C. Zayfert, and R.T. Gross, *The Pain Anxiety Symptoms Scale: development and validation of a scale to measure fear of pain.* Pain, 1992. **50**(1): p. 67-73.
13. McCracken, L.M. and L. Dhingra, *A short version of the Pain Anxiety Symptoms Scale (PASS-20): preliminary development and validity.* Pain Res Manag, 2002. **7**(1): p. 45-50.
14. McCracken, L.M., K.E. Vowles, and C. Eccleston, *Acceptance-based treatment for persons with complex, long standing chronice pain: a preliminary analysis of treatment outcome in comparison to a waiting phase.* Behavior Research and Therapy, 2005. **43**: p. 1335-1346.
15. McCracken, L.M., K.E. Vowles, and C. Eccleston, *Acceptance of chronic pain: component analysis and a revised assessment method.* Pain, 2004. **107**: p. 159-166.
16. *ECDEU Assessment Manual for Psychopharmacology*, ed. W. Guy. 1976, Rockville, MD: US Department of Health, Education, and Welfare Public Health Service Alcohol, Drug Abuse, and Mental Health Administration.
17. Greco, C.M., L. Yu, K.L. Johnston, N.E. Dodds, N.E. Morone, R.M. Glick, et al., *Measuring nonspecific factors in treatment: item banks that assess the healthcare experience and attitudes from the patient's perspective.* Qual Life Res, 2016. **25**(7): p. 1625-34.
18. Rolke, R., R. Baron, C. Maier, T.R. Tolle, D.R. Treede, A. Beyer, et al., *Quantitative sensory testing in the German Research Network on Neuropathic Pain (DFNS): standardized protocol and reference values.* Pain, 2006. **123**(3): p. 231-243.
19. Kabat-Zinn, J., *An outpatient program in behavioral medicine for chronic pain patients based on the practice of mindfulness meditation: theoretical considerations and preliminary results.* Gen Hosp Psychiatry, 1982. **4**(1): p. 33-47.
20. Strigo, I.A., S.C. Matthews, and A.N. Simmons, *Decreased frontal regulation during pain anticipation in unmedicated subjects with major depressive disorder.* Translational Psychiatry, 2013. **3**(3): p. e239.
